# First person – Sukalp Muzumdar

**DOI:** 10.1242/dmm.045146

**Published:** 2020-05-29

## Abstract

First Person is a series of interviews with the first authors of a selection of papers published in Disease Models & Mechanisms, helping early-career researchers promote themselves alongside their papers. Sukalp Muzumdar is first author on ‘Genetic activation of Nrf2 reduces cutaneous symptoms in a murine model of Netherton syndrome’, published in DMM. Sukalp conducted the research described in this article while a PhD student in the lab of Prof. Dr Sabine Werner and PD Dr Matthias Schäfer at ETH Zurich, Zurich, Switzerland. He is now a postdoctoral fellow in the lab of Jesse Gillis at Cold Spring Harbor Laboratory, Cold Spring Harbor, NY, USA, investigating the mechanisms behind rare diseases and exploring avenues for novel treatments.


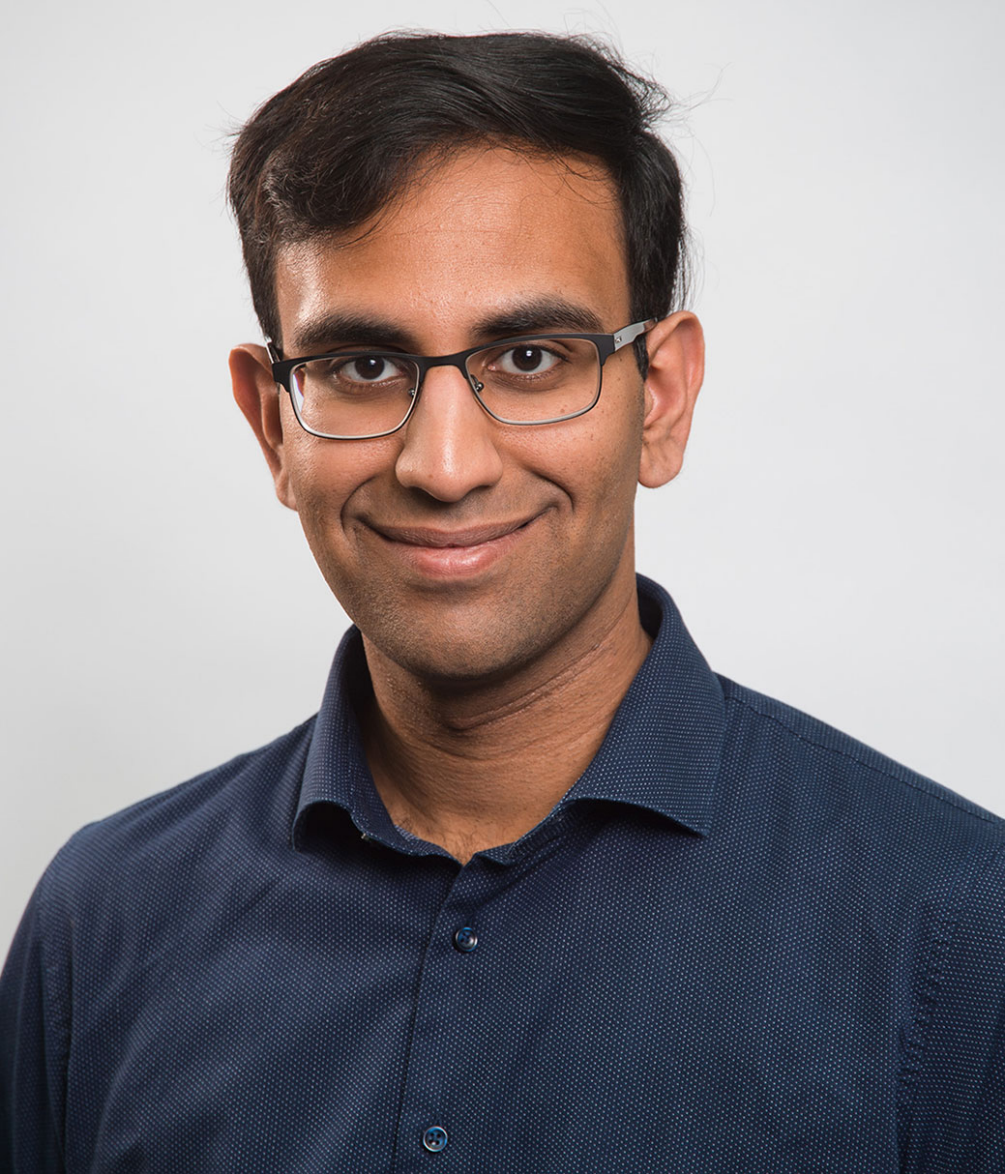


**Sukalp Muzumdar**

**How would you explain the main findings of your paper to non-scientific family and friends?**

Animal, and especially mouse, models constitute a cornerstone of biological research. Specifically, in rare diseases, where access to patients and human samples can be a limiting factor, animal models can fill the need for a testbed for novel drugs and treatments. One such rare disease that currently lacks effective therapies is Netherton syndrome. Patients suffering from this disease have a mutation in a gene (*SPINK5*) that is essential for the skin's barrier function, which plays an indispensable role in protecting the body from infections and dehydration. Netherton syndrome patients suffer from recurrent infections and allergies, as well as from dry and itchy skin. In the research described here, we were able to show that the activation of the cytoprotective protein Nrf2, which is known to be crucial in detoxifying and protecting cells from oxidative stress, surprisingly led to a significant amelioration of disease symptoms in a mouse model of Netherton syndrome. Mice with Nrf2 activation exhibited significantly better barrier function, as well as a reduction in inflammatory parameters in the skin. This improvement was most likely driven by an Nrf2-mediated re-balancing of the enzyme-inhibitor network operating at the outermost layers of the skin by increasing the expression of a specific enzyme inhibitor (Slpi). We were able to show that this increased expression of SLPI occurred in human cells as well upon NRF2 activation, demonstrating the clinical translatability of our results.

**What are the potential implications of these results for your field of research?**

Netherton syndrome remains hard to manage clinically, with no targeted therapies available on the market. Additionally, patients have a poor quality of life due to frequent skin infections and a large variety of food and environmental allergies. Our research may provide the basis for the development of a novel therapeutic strategy for the clinical management of Netherton syndrome, thus helping this underserved population. It also establishes NRF2 as an important target for the treatment of other skin diseases, which involve a similar defect in barrier function.

**What are the main advantages and drawbacks of the model system you have used as it relates to the disease you are investigating?**

The primary advantages of the model system we have used is that it recapitulates the genetics and the primary symptoms of the human disease and is readily available from various mouse model repositories. Furthermore, murine skin and immune systems have many similarities to humans and thus mice are a good model for this class of diseases. There are, however, several drawbacks to this mouse model, including the fact that the mice exhibit a much stronger phenotype than human patients, possibly due to compensatory mechanisms in humans. Furthermore, we were able to activate Nrf2 only in the skin due to the promoter used in this study, and a broader activation may potentially be required for a clinical response in Netherton syndrome.

**Describe what you think is the most significant challenge impacting your research at this time and how will this be addressed over the next 10 years?**

The most significant challenge in working with rare diseases is the paucity of research material and human subjects. Additionally, very often, patient registries (if they exist) are poorly maintained, and thus make access to important biological samples challenging. Thus, the development and characterization of animal models will remain to be of the utmost importance over the next 10 years to serve as a pre-clinical testbed for novel therapeutics. Another important upcoming solution is the development of organoid and organotypic cultures, which are derived from patient material and can also be exploited for the development of treatments for rare diseases.

**Figure:**
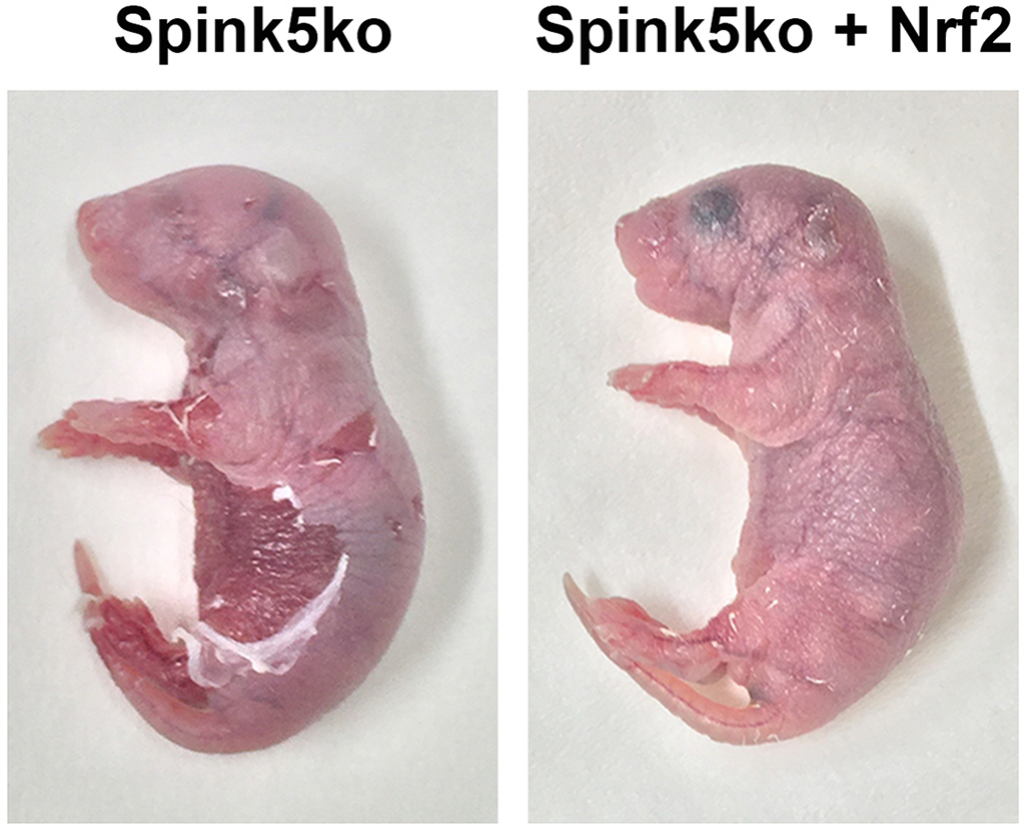
**Photo showing a mouse model of Netherton syndrome (Spink5ko mice) at birth without Nrf2 activation (left) and with Nrf2 activation (right).**

**What changes do you think could improve the professional lives of early-career scientists?**

I think it is important to ensure that early-career scientists in biology are trained in entrepreneurship and product development to ensure that they understand the path towards the commercialization of their research results. I also believe such training helps foster better academic-industry collaboration and communication and helps create opportunities for early-career scientists to translate their findings into tangible products. Furthermore, research is an intrinsically collaborative endeavor, and I think that further encouraging inter- and cross-disciplinary research at a grassroots level would help early-stage researchers to successfully pursue academic careers.

“[…] encouraging inter- and cross-disciplinary research at a grassroots level would help early-stage researchers to successfully pursue academic careers.”

**What's next for you?**

I am currently working as a postdoctoral fellow at Cold Spring Harbor Laboratory as a part of the group of Jesse Gillis, where I am involved in studying the pathogenesis of rare blood disorders and also working towards discovering novel drug targets. My long-term goals are to apply for a tenure-track position in academia.
